# Refraction and pupil diameter in 3-year- and 1-month-old children as measured by Spot Vision Screener

**DOI:** 10.1038/s41598-019-51993-1

**Published:** 2019-10-30

**Authors:** Shunya Tatara, Fumiatsu Maeda, Nobuko Mizuno, Atsushi Noguchi, Kiyoshi Yaoeda, Haruki Abe

**Affiliations:** 1 Department of Orthoptics and Visual Sciences, Niigata University of Health and Welfare, Niigata, Niigata Japan; 2 Field of Visual Sciences, Graduate School Niigata University of Health and Welfare, Niigata, Niigata Japan; 3 Department of Ophthalmology, Fujieda Municipal General Hospital, Fujieda, Shizuoka Japan; 4 Department of Ophthalmology, Yaoeda Eye Clinic, Nagaoka, Niigata Japan; 5 Division of Ophthalmology and Visual Sciences, Niigata University Graduate School of Medical and Dental Sciences, Niigata, Niigata Japan

**Keywords:** Physical examination, Risk factors

## Abstract

Spot Vision Screener (SVS) can conduct refraction tests for both eyes within a short period. This study aims to evaluate the refraction and pupil diameters of 3-year- and 1-month-old Japanese children using SVS in regular medical checkup. We examined 2438 eyes of 1219 children (age: 3-year- and 1-month) in Fujieda (Shizuoka, Japan) to assess their refraction and pupil diameters and eye-position screening conducted by SVS. SVS successfully measured 1217 children (99.8%). Regarding the right eye refraction, the spherical power was +0.70 ± 0.55 D (median, +0.75 D), and the cylindrical power was −0.67 ± 0.49 D (median, −0.50 D). The pupil diameter of the right eyes was 5.57 ± 0.79 (median, 5.60) mm. we could obtain a large number of basic data for 3-year- and 1-month-old Japanese children. However, refraction and pupil diameter of children were not normally distributed, so careful handling of children’s basic data on the eye is necessary.

## Introduction

Amblyopia is a failure of vision to functionally develop without normal visual acuity despite an accurate prescription for refractive correction and the absence of any organic abnormality in the eye. The major causes for amblyopia include strabismus, form vision deprivation, and refractive error^1^. Occasionally, visual acuity is not completely developed if amblyopia is inadequately treated during the sensitive period for vision. From this standpoint, the early detection and treatment of amblyopia is crucial^1,2^. For the early detection of amblyopia, vision test has been included in the medical checkup of 3-year-old children (checkup) in Japan since 1990^3^. From the refraction test validity to enhanced checkup accuracy^4^, the number of cities implementing the objective refraction test has increased, with the result that its usefulness has been reported^5^.

To date, various devices have been used for the objective refraction test in checkups^6^. Jorge *et al*.^7^ reported that retinoscopy is more accurate than automatic refraction when performed by an experienced clinician, offering a better starting point to noncycloplegic refraction. However, the limitation of retinoscopy is its precision relies on the skills and, thus, inter-observational differences in the measurers; automatic refraction is more independent of such differences among limitations such as low accommodation removal effect and that subjects must be seated. Conversely, both eyes can be measured within a short period of visual fixation, as eyes are opened and are very close to the natural status in photorefraction devices^8^, making it readily applicable in checkups for its efficacy. Spot Vision Screener (SVS; Welch Allyn, NY, US), introduced in Japan by Welch Allyn in 2015, is a portable refraction-measuring device based on off-axis type photorefraction^9^. SVS records images of both pupils simultaneously and generates information on noncycloplegic refractive status, pupil size, pupillary distance, and ocular deviation in real time. Regarding the measurement of “Amblyopia Risk Factor” (ARF) by SVS, sensitivity has been reported to be 87–92.6%, specificity as 74–90.6%, and good values have been displayed in the previous reports^10–12^.

The referral criteria of SVS is based on the ARF standard prescribed by the American Association for Pediatric Ophthalmology and Strabismus^12^. Nonetheless, Sravani *et al*.^13^ reported the use of an ethnicity- or individual-specific defocus calibration factor for the precise estimation of refraction using photorefraction.

In addition, Ra *et al*.^14^ emphasized the low reliability of checkup when examining Japanese subjects using a foreign database; nevertheless, neither fundamental data nor sufficient consideration exist for the refraction test of Japanese subjects. Furthermore, inadequate data exist of 3-year-old children, compared with extensive studies on the pupil diameter of adult subjects^15^. Although Tabuchi *et al*.^16^ reported that the average normal pupil diameter of healthy 3-year-old children was 5.4 ± 0.5 mm, they examined 5 cases only. Hence, this study aims to investigate the efficacy of SVS, and to evaluate refraction degrees, and pupil diameters of 3-year-old children in checkups with a huge population.

## Results

### SVS measurement possibility rate

We observed a valid measurement in 1217 of 1219 subjects (99.8%). Two invalid measurements were with one pupil constriction smaller than the lower limit of 4 mm when measuring the diameter and another was without impossible visual fixation during 1 s.

### The refraction measured by SVS

Of the 1217 subjects who underwent SVS measurement, 61 (5.0%) were suspected with abnormal refraction, 6 (0.5%) with abnormal eye position, and 4 (0.3%) with both abnormal refraction and eye positions, making up 71 (5.8%) workup cases overall. In addition, 52 eyes of 32 subjects had hyperopia of over +2.00 D, 56 eyes among 44 children had astigmatism of over +2.00 D, 4 eyes among 3 children had myopia of >1.50 D, and 12 children had anisometropia of over +2.00 D. Of the 71 subjects who required detailed examination, 69 were able to confirm their visit to the medical institution. Of the 69 patients, 64 (92.8%) were diagnosed as requiring continuous hospital visits.

**Figure 1:**
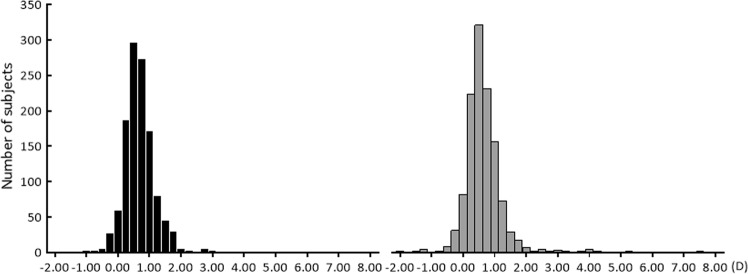
The histogram of the spherical power. The spherical power of 3-year and 1-month-old children measured by Spot Vision Screener (SVS). Black, the right eye; gray, the left eye. Spherical power didn’t follow the normal distribution for both the right and left eyes (the right eye; *P* < 0.01, the left eye; *P* < 0.01, Kolmogorov–Smirnov test).

**Figure 2:**
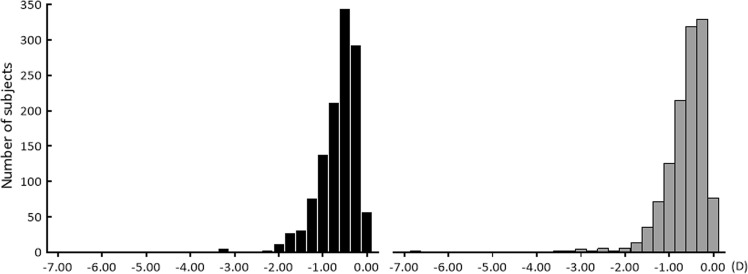
The histogram of the cylindrical power. The cylindrical power of 3-year and 1-month-old children measured by Spot Vision Screener (SVS). Black, the right eye; gray, the left eye. Cylindrical power didn’t follow the normal distribution for both the right and left eyes (the right eye; *P* < 0.01, the left eye; *P* < 0.01, Kolmogorov–Smirnov test).

Regarding the refraction of the right eyes of 1207 subjects with valid result and measurement, the spherical power was +0.70 ± 0.55 D (median, +0.75 D). The average cylindrical power was −0.67 ± 0.49 D (median, −0.50 D). The average spherical diopter power of the left eye was +0.64 ± 0.61 D (median, +0.50 D; Fig. 1). Furthermore, the average cylindrical power was −0.62 ± 0.50 (median, −0.50 D; Fig. 2). Spherical power didn’t follow the normal distribution for both the right and left eyes (the right eye; *P* < 0.01, the left eye; *P* < 0.01, Kolmogorov–Smirnov test). The skewness of the right eye was 1.62, and the kurtosis was 9.48. The skewness of the left eye was 2.79, and the kurtosis was 21.8. Cylindrical power didn’t follow the normal distribution for both the right and left eyes (the right eye; *P* < 0.01, the left eye; *P* < 0.01, Kolmogorov–Smirnov test). The skewness of the right eye was −2.01 and the kurtosis was 7.07. The skewness of the left eye was −3.04 and the kurtosis was 22.4. When comparing the spherical power of the left and right eyes, anisometropia was 0.29 ± 0.37 D (median, 0.25 D; maximum value = 4.50 D).

### Pupil diameter

**Figure 3:**
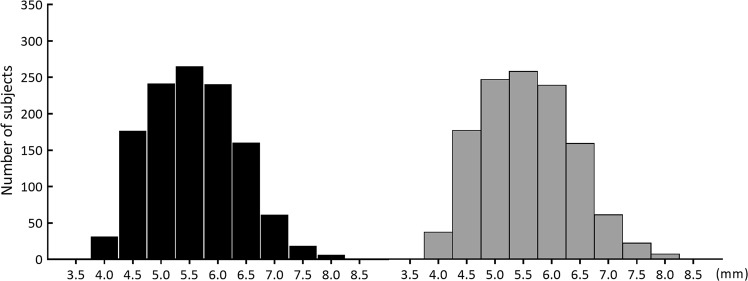
The histogram of the pupil diameter. The pupil diameter of 3-year and 1-month-old children measured by Spot Vision Screener (SVS). Black, the right eye; gray, the left eye. Pupil diameter didn’t follow the normal distribution for both the right and left eyes (the right eye, *P* < 0.01; the left eye, *P* < 0.01, Kolmogorov–Smirnov test).

In this study, the pupil diameter of the right eyes was 5.57 ± 0.79 (median, 5.60) mm, and that for the left eyes was 5.56 ± 0.80 (median, 5.50) mm (Fig. 3). We observed no difference between the pupil diameter of the left and right eyes (*P* = 0.104, Wilcoxon signed-rank test). Pupil diameter didn’t follow the normal distribution for both the right and left eyes (the right eye, *P* < 0.01; the left eye, *P* < 0.01, Kolmogorov–Smirnov test). The skewness of the right eye was 0.30, and the kurtosis was −0.35. The skewness of the left eye was 0.32, and the kurtosis was −0.36.

## Discussion

Like Fujieda, the Shizuoka Prefecture introduced SVS for checkups of 3-year and 1-month-old children in 2016, which was the earliest in Japan, enabling us to attain a large pool of data with 1217 Japanese subjects. In this study, the SVS checkup measurement possibility rate was 99.8%, implying that almost all children underwent the SVS test. Thus, we attained an unprecedentedly large data pool with refraction and pupil diameter data of 3-year-old Japanese children, which was extremely precious.

The success rate was 99.8%, with only two invalid measurements, and it was the highest in comparison with all previous refraction tests^17–19^ (Table 1). As the distance between subjects and the device was 1 m, the measurement was performed without any psychological burden on children. Likewise, previous studies^14,20–22^ investigating the SVS measurement of 6-month to 6-year-old children reported the possibility rates at 89.7–100%^14,20–22^ (Table 2). Moreover, Forcina *et al*.^21^ reported a possibility rate of 89.7% with subjects aged <3 years (average: 23.3 months). Even with the large pool of 1,219 subjects, some previous studies^14,20–22^ revealed a similarly high possibility rate. Compared with other refraction tests, SVS can be conducted on lower age groups, making it appropriate in the screening of checkups.

**Table 1:** Reports About Refraction Tests on 3-year-old Children Through Other Devices.

Author	Years	Measuring method	*n* (eyes)	Measurement possibility rate (%)	Spherical power ± SD (D)	Anisometropia of 2.00 D above (%)	Astigmatism of 2.00 D above (%)
Kanda *et al*.	1986	Retinoscopy	665 (1330)	95.8	+0.22 ± 0.67	0.6	1.4
Tamaki *et al*.	1990	Table-mounted auto refractometer	459 (905)	>90 (no concrete figure)	+0.33 ± 0.95	2.6	1.8
Saito *et al*.	2010	Portable refractometer	10,454 (20,908)	98.3	−0.74 ± 1.16	3.3	1.6
This study	2018	SVS	1217 (2434)	99.8	+0.67 ± 0.59	1.0	2.3

SVS: Spot Vision Screener.

**Table 2:** Reports About SVS Measurement Possibility Rate in Small Children.

Author	Years	target age	measurement possibility rate (%)	*n* (people)
Arnold *et al*.	2014	47 months (1–12 years)	96	108
Blake *et al*.	2017	23.3 months (6–35 months)	89.7	184
Suzuki *et al*.	2017	3 years and 6 months	100	71
Ra *et al*.	2017	0–2 year	96.6	58
″		3 year	98.2	55
″		4–6 year	100	113
This study	2018	3 years and 1 month	99.8	1,219

SVS: Spot Vision Screener.

The spherical power of the right eyes of non-accommodation paralysis in 3-year and 1-month-old children, as measured by SVS (+0.70 ± 0.55 D), was similar to the results of a series of studies from previous checkups in Japan^17–19^ (Table 1). Kanda *et al*.^17^ reported that the spherical power of non-accommodation paralysis in 3-year-old children was +0.22 ± 0.67 D; the refraction of 3-year-old children was estimated as +1.00 D to +1.50 D considering the impact of the accommodation on the refraction as some subjects were far-sighted with an average of 1.33 D in mydriasis.

Compared with the spherical power of non-accommodation paralysis reported in 3-year-old children in the literature, the results in this study were more hyperopic. While retinoscopy is associated with minimal accommodation effects, Nishida *et al*.^23^ reported no significant differences between the spherical power measured by retinoscopy and SVS. Nevertheless, the spherical power in this study was higher than that of far-sighted children (age: 3 years) as measured previously using retinoscopy, which could be attributed to SVS mechanical features that detected a more hyperopic value. Another possibility is the shift from the average of the spherical power in 1986 when the retinoscopy study was conducted; the current average could be, in fact, different.

In this study, 56 eyes of 44 children (2.3%) reported an astigmatic vision of over +2.00 D; the proportion was relatively high compared with the previous studies^17–19^ (Table 1). In previous reports, high cylindrical power was obtained under the influence of the inclination of the SVS device or the face of subjects^24^; it remains high when compared with retinoscopy; this is probably because of SVS mechanical features.

We observed anisometropia of +2.00 D in 12 subjects (1.0%). Compared with the rate of anisometropia rate reported previously^17–19^ (Table 1), SVS is lower than the portable and table-mounted refraction types but it remained the same when compared with retinoscopy. Saito *et al*.^19^ highlighted that the refraction difference between the two eyes using the portable device could be too large because of the accommodation tension remained after the test on one side. Meanwhile, as tests on both opened eyes were conducted simultaneously in SVS, no influence was noted on either side, which is attributed to lower SVS’ anisometropic rate than other refraction-measuring devices.

Regarding the pupil diameter, several studies have examined about adult subjects; however, limited data are available about children. Although Tabuchi *et al*.^16^ reported that the average normal pupil diameter of healthy 3-year-old children was 5.4 ± 0.5 mm, they examined only 5 cases; moreover, they took pictures of the pupil manually using a pupil-specific polaroid camera right under the pupil, thereby lowering the accuracy and the close distance affected the pupil contraction.

Ikeda *et al*.^25^ highlighted that the pupil diameter of immature children is smaller than that of healthy children, while it is not much necessarily convincing for the small control group of just six cases; as they measured using infrared radiation electron-measuring goggles, the accuracy was high, however because googles were needed in the measurement, it rather deviated from standard vision sights.

In this study, although SVS measurement was conducted in a semi-dark room, the distance between subjects and the device was as far as 1 m, with no blocking in the subjects’ vision, making it highly close to normal, daily eyesight. In addition, the pupil diameter was evaluated by infrared radiation vision analysis in electron-measuring devices, rendering the highy accuracy of the measurements. The right pupil diameter of 3-year and 1-month-old children was 5.57 ± 0.79 mm, which corroborated that the finding reported by Tabuchi *et al*.^16^.

Notably, avoiding interference when conducting a refraction test on children is imperative. It is commonly believed that retinoscopy is ideal because of its minimal accommodation influence. In this study, the spherical power was measured at the far position beyond the far-sighted data measured by traditional retinoscopy. In addition, as the measurement was performed on bilaterally opened eyes, the impact on anisometropia was considered to be minimal. Besides, the measurement possibility rate was high compared with conventional methods; in SVS, measurement was performed on both sides simultaneously in 1 s of visual fixation, which is valuable in checkups. However, the large cylindrical power merits attention. Furthermore, the data in this study were obtained from non-accommodation paralysis refraction data; thus, comparisons with accommodation paralysis data is warranted in further studies.

Because the measurable rate for SVS in the health examination was 99.8%, we were able to obtain a large number of basic data for 3-year- and 1-month-old Japanese children. The results of the present study can be basic data, such as refraction and pupil diameter. However, refraction and pupil diameter of children are not normally distributed, so careful handling of these data is necessary. As a result of the screening using SVS, 5.8% of children required detailed examination because of refractive error and abnormal eye position. As a result of detailed examination, 92.8% of children were diagnosed as requiring continuous hospital visits. This indicates that the SVS false positive rate for 3-year- and 1-month-old using the Fujieda’s criteria is low.

## Methods

We examined 2438 eyes of 1219 children undergoing checkup in Fujieda between April 2016 and March 2017 for their eye position, pupil diameter, and refraction measured by orthoptists from the Fujieda Municipal General Hospital (Fujieda, Japan) using SVS in a semi-dark room (4 lux). The population data of Fujieda City shows that as of March 31, 2016, 1265 children were eligible for the checkup in Fujieda for the 3-year- and 1-month-old during the year starting April 1, 2017. It is unknown how many children moved in or out of the city during the same period. Since 1219 of the 1265 eligible children took the checkup, the checkup rate is calculated to be 96.4%. We compared the SVS checkup possibility in this study with those of other refraction measurements. SVS is held approximately 1 m from the subject while the children look at the display of twinkling lights and sounds. SVS reports whether the subject is too far or too close and shows a spring circle and the child’s face when data acquisition is occurring. Data acquisition is usually complete in approximately 1 s.

Fujieda City determined the criteria required for the detailed examination based on the previous research in Shizuoka City^5^ in which hyperopia, myopia, and anisometropia were classified according to the spherical power. We evaluated the number of children needing workups by combining the SVS measurement results with Fujieda’s criteria, which defines abnormal refraction as hyperopia ≧2.00 D, myopia ≧1.50 D, astigmatism ≧2.00 D, and anisometropia ≧2.00 D; abnormal eye position is defined as having the extropia, upper or lower manifest deviation ≥8° and esotropia manifest deviation of ≥5°. Furthermore, we defined abnormal pupil difference as having ≥1-mm difference between the left and right eyes. These values as a marker of defining abnormal eye position and pupil difference are reference values recommended by SVS^12^.

Excluding 10 subjects with abnormal eye position from 1217 subjects with valid results, we analyzed the refraction of both eyes and pupil diameter in the remaining 1207 subjects. In addition, the SVS measurement success rate was compared with those reported in previous checkups. Of these, a single eye with hyperopia was above +7.50 D; as it was the measurement limit of SVS, the refraction was evaluated as +7.50 D.

This study protocol was approved by the Ethics Committee of Niigata University of Health and Welfare (17892–170914) and adhered to the tenets of the Declaration of Helsinki. Approval by the Ethics Committee was set as a substitute for informed consent by placing a poster that detailed the purpose and contents of this research at the examination venue. In this study, since the subject was under 18 years old, we got informed consent from parent or legal guardian. A two-sided test was used for statistical significance, and the significance level was set at p < 0.05.
